# The Effect of Manufacturing Agglomeration on Haze Pollution in China

**DOI:** 10.3390/ijerph15112490

**Published:** 2018-11-08

**Authors:** Jun Liu, Yuhui Zhao, Zhonghua Cheng, Huiming Zhang

**Affiliations:** 1 China Institute of Manufacturing Development & School of Management Science and Engineering, Nanjing University of Information Science & Technology, Nanjing 210044, China; chengzhonghua0106@163.com (Z.C.); 002068@nuist.edu.cn (H.Z.); 2 School of Business, Nanjing University of Information Science & Technology, Nanjing 210044, China; 20161263742@nuist.edu.cn

**Keywords:** manufacturing agglomeration, haze pollution, dynamic spatial panel model, regional differences

## Abstract

Based on panel data on 285 Chinese cities from 2003 to 2012, we use a dynamic spatial panel model to empirically analyze the effect of manufacturing agglomeration on haze pollution. The results show that when economic development levels, population, technological levels, industrial structure, transportation, foreign direct investment, and greening levels are stable, manufacturing agglomeration significantly aggravates haze pollution. However, region-specific analysis reveals that the effects of manufacturing agglomeration on inter-regional haze pollution depends on the region: the effect of manufacturing agglomeration on haze pollution is the largest in the Western region, followed by the Central region, and is the least in the Eastern region. Based on the above conclusions, we put forward several specific suggestions, such as giving full play to the technology and knowledge spillover effects of manufacturing agglomeration, guiding manufacturing agglomerations in a scientific and rational way, accelerating the transformation and upgrading of manufacturing industries in agglomeration regions.

## 1. Introduction

Since reform and opening up, industrialization and urbanization in China has rapidly advanced. At the same time, those activities associated with manufacturing agglomeration have also developed rapidly. As it is the backbone of the economy, manufacturing is the industry most typically associated with agglomerations and this is no less true in China. Manufacturing agglomeration promotes regional economic development and enhances its competitiveness through, not only an agglomeration effect, but also a correlation effect and a spillover effect. Although manufacturing agglomeration promotes regional economic development, it is, however, often accompanied by widespread pollution including, amongst other things, pollution of rivers and haze. Since 2013, the major cities of China have been repeatedly shrouded in haze pollution of long duration and wide range. The haze problem has seriously affected both people’s physical and mental health and daily life. According to the 2016 China Environmental Status Bulletin, only 84 cities reached the ambient air quality standard in 2016. In fact, the air quality standard was met by less than a quarter of all cities. Not only was PM_2.5_ the primary pollutant, but it accounted for 80.3% of the total pollution days designated high PM_2.5_ days. Faced with this serious problem of urban haze pollution, the Chinese government is taking action. In the ten goals of the “13th Five-Year” plan, China, for the first time, is incorporating a strengthening of ecological projects into its five-year plan, which shows that the development of the environment occupies an important position in the future economic and social planning of our country. The 19th National Congress of the CPC also clearly stated that it was necessary to focus on solving certain outstanding environmental problems, especially the prevention and control of atmospheric pollution.

Although the formation of haze may be due to terrain or meteorology, the main reasons for the formation of haze pollution are irrational industrial structure [1], rapid urbanization and industrialization [2], vehicle exhaust emissions [3] and other human or economic factors. Furthermore, there are regional differences in the spatial distribution of haze pollution that are quite obvious. The areas of high-frequency haze pollution in China are mainly distributed in the Beijing-Tianjin-Hebei region, and the Yangtze River Delta [4], the very areas that are the principal areas of China’s manufacturing agglomerations. We cannot help asking whether manufacturing agglomeration has aggravated haze pollution and by how much? Up to now, few scholars have studied the relationship between occurrence and effects of haze pollution from the perspective of manufacturing agglomeration. This is a key issue to be discussed and studied in this paper. The rest of this article is arranged as follows: Section 2 is a review of the literature; Section 3 introduces the model specifications and explains the variable and data sources; Section 4 describes empirical results and discussion; and Section 5 draws conclusions and provides some recommendations.

## 2. Literature Review

There are few studies on the effect of manufacturing agglomeration on haze pollution, and the research that has been done has mainly focused on the effects of industrial agglomeration on environmental pollution. Conclusions can be divided into the following three categories. In the first category, it is postulated that industrial agglomeration aggravates environmental pollution and the main reasons are summarized as follows. First, pollution increases dramatically with both the expansion of industrial scale and increases in output that come with industrial agglomeration and this leads to an aggravation of any existing environmental pollution. Second, in order to attract more investment, some underdeveloped regions lower their environmental regulation standards; the agglomeration of a large number of pollution-intensive enterprises in a certain area will aggravate local environmental pollution. Finally, in order to save costs, many enterprises are often characterized by a “free rider” mentality; they are unwilling to take responsibility for improving environmental pollution in the agglomeration regions, which then leads to even further environmental degradation.

Verhoef and Nijkamp used a general spatial equilibrium model to analyze the effect of industrial agglomeration on environmental pollution and found that industrial agglomeration did indeed aggravate environmental pollution [5]. Zhang and Wang empirically analyzed the interaction between economic agglomeration and environmental pollution; their results showed that economic agglomeration aggravated environmental pollution but environmental pollution restrained economic agglomeration [6]. Sun and Yuan used prefecture-level data to empirically analyze the relationship between industrial agglomeration and environmental pollution; the study showed that, although manufacturing agglomeration aggravated environmental pollution, service agglomeration was conducive to improving it [7]. Liu et al. used the spatial panel data to empirically study the impact of industrial agglomeration on environmental pollution. The results showed that, in China, industrial agglomeration significantly aggravated environmental pollution [8]. Liu et al. empirically analyzed the effect of industrial agglomeration on environmental pollution. Their study found industrial agglomeration does exacerbate the level of industrial pollution although there was a weakening of the negative environmental effects [9].

The second view is that industrial agglomeration improves environmental pollution and the main reasons are summarized as follows. First, with the continuous development and expansion of areas of industrial agglomeration, the scale effect of industrial agglomeration is beneficial to the centralized management of pollutants and actually improves the efficiency of disposal of pollutants and waste. Thus, pollution treatment scales of economy are realized. Second, industrial agglomeration will not only bring specialization in the industrial division of labor but the cooperation between upstream and downstream enterprises is also conducive to the recycling of pollutants and encourages the formation of a recycling economy. Finally, industrial agglomeration is conducive to both knowledge spillover and technological innovation. This may stimulate enterprises into improving production technology levels and adopting green production technologies that can reduce pollutant emissions, thereby abating environmental pollution in agglomeration areas.

Hosoe and Naito confirmed industrial agglomeration was conducive to both improving the level of environmental technology in the region as well as promoting knowledge spillover, thereby abating environmental pollution in the agglomeration areas [10]. The research by Chen and Hu showed that industrial agglomeration can cause competition among enterprises of the region and is thus both beneficial to improvements in production technology in enterprises and reduces pollutant emissions [11]. Zeng and Zhao constructed a mathematical model consisting of two countries and two departments to study the effect of manufacturing agglomeration on environmental pollution. The results showed that manufacturing agglomeration was conducive to improving environmental pollution [12]. Lu and Feng found that, in China, increases in the degree of industrial agglomeration are conducive to reductions in the intensity of industrial pollution which leads to an overall reduction in environmental pollution [13]. Jiang and Zheng found that the degree of environmental pollution in urban areas with high levels of industrial agglomeration was relatively low [14].

The third view is that there is a non-linear relationship between industrial agglomeration and environmental pollution. This may be because the effect of industrial agglomeration on environmental pollution is different at different stages of development. When industrial agglomeration is in the growth stage, enterprises focus more on economic benefits and so continuously expand production. With insufficient emphasis on environmental protection, the quality of the environment deteriorates. When the industrial agglomeration further develops, the spillover effect of knowledge and technology in the agglomeration area becomes obvious, both division of labor and cooperation are expanded and the cost of pollution treatment is reduced. The end result is an improvement in the environment of the agglomeration area.

Many studies have shown an inverted “U” relationship between pollution and growth. Luo et al. found that there was a nonlinear relationship between industrial agglomeration and nitrogen dioxide and that the concentration of atmospheric pollutants in the provincial capital cities showed an inverted “U” relationship with economic growth [15]. He et al. studied the relationship between economic agglomeration and environmental pollution. The results showed that there was an inverted “U” relationship between economic density and industrial sulfur dioxide [16]. A non-parametric augmented regression model was used by Xu and Lin to analyze the effect of industrial agglomeration on carbon dioxide emissions in China and the results showed that there was an inverted “U” relationship between industrial agglomeration and carbon dioxide emissions [17]. Li et al. applied a threshold regression method to empirically test the impact of industrial agglomeration on environmental pollution. They found that there was an inverted “U” relationship between industrial agglomeration and environmental pollution [18]. According to the research of Yuan and Xie, industrial agglomeration and environmental pollution showed an inverted “U” relationship, and technological innovation had an important influence on the position of the “turning point” in the relationship [19].

Previous studies have achieved plentiful results, but there are still some other aspects to be studied. First, in the field of economics, when studying haze scholars mostly study the relationship between the occurrence and the role of haze from the perspective of urbanization, population, industrial structure, and energy structure; there are few papers on haze pollution from the perspective of manufacturing agglomeration. Second, most of the existing literature is based on ordinary panels or dynamic panels, ignoring the spatial spillover effects and dynamic effects of haze pollution and so leading to errors in estimation and analysis [20]. Third, most of the existing studies use provincial-level data for analysis but, in China, there are few studies that use city-level data. China’s provinces are spatially large and exhibit significant internal differences so it is difficult to accurately capture the spatial spillover effect of haze pollution [21]. Based on existing research, we use the 2003 to 2012 panel data on 285 prefecture-level and above cities and employ dynamic spatial panel models to study both the effect of manufacturing agglomeration on haze pollution and its regional differences.

## 3. Model Specification, Variables Description, and Data Sources

### 3.1. The Establishment of a Spatial Econometric Model

Ehrlich and Holdren [22] proposed the IPAT environmental pollution model and compartmentalized the environmental pressure into three parts, namely, I=P×A×T, where I, P, A and T denote the environmental pressure, population, economic development level and technical level, respectively. Although the IPAT model is both widely recognized and applied due to its simplicity and effectiveness in analyzing environmental impacts, there are shortcomings in linear equivalence between variables. Dietz and Rosa [23] proposed the STIRPAT model based on the IPAT model, which retained the original model and also incorporated a random item to facilitate empirical analysis. The basic form of the STIRPAT model is: $I_{it}=\alpha \times P_{it}^{\theta }\times A_{it}^{\gamma }\times T_{it}^{\varphi }\times \varepsilon_{it}$, where *i* represents region, *t* represents time, α denotes the constant term, θ, γ, φ are estimated parameters of *P*, *A* and *T*, respectively, and ε is a random error term. Based on the STIRPAT model, this paper introduces a manufacturing agglomeration variable to analyze its impact on environmental pressure, and extends the STIRPAT model as follows:

(1) $$I_{it}=\alpha \times P_{it}^{\theta }\times A_{it}^{\gamma }\times T_{it}^{\varphi }\times Agglo_{{}_{it}}^{\beta }\times \varepsilon_{it}$$

where ${Agglo}_{it}$ denotes the degree of manufacturing agglomeration, and β denotes the elasticity of the concentration of manufacturing agglomeration on environmental pressure. Combined with the previous research in the literature, we take logarithms on both sides of the model (1) to eliminate the heteroscedasticity between variables, this paper establishes the following general panel data model based on model (1):

(2) $$\ln I_{it}=\ln\alpha +\beta \ln{Agglo}_{it}+\theta \ln P_{it}+\gamma \ln A_{it}+\varphi \ln T_{it}+\delta \ln X_{it}+\ln\varepsilon_{it}$$

In this paper, we use the haze pollution concentration to represent environmental pressure (*I*). X is the control variable that affects haze pollution. According to the relevant literature, this paper selects the industrial structure (Stru), foreign direct investment (FDI), transportation (Trans), greening level (Green) and central heating (Heat) as control variables. Haze pollution not only occurs in individual regions but is also concentrated in some areas. This indicates that haze pollution has spillover effects and that there are spatial interactions between cities [24,25]. Moreover, haze pollution has an obvious path dependence characteristic. In view of this, this article incorporates its first-order lag and spatial lag into model (3), which not only considers the spatial effect of haze pollution but also examines the effect of early pollution factors on this period. The model is as follows:

(3) $$\begin{array}{l} \ln I_{it}=\tau \ln I_{i,\left(t-1\right)}+\rho \sum_{j=1}^{N}W_{ij}\ln I_{jt}+\ln\alpha +\beta \ln{Agglo}_{it}+\gamma \ln{Pgdp}_{it}+\\ \theta \ln{Pop}_{it}+\varphi \ln{Tech}_{it}+\delta \ln X_{it}+\eta_{i}+\nu_{t}+\varepsilon_{it}\\ \varepsilon_{it}=\lambda \sum_{j=1}^{N}W_{ij}\varepsilon_{jt}+\mu_{it} \end{array}$$

where Pgdp, Pop, Tech respectively denote economic development level (*A*), population (*P*) and technical level (*T*). τ represents the regression coefficient of the first-order lag of haze pollution, reflecting the effect of previous related factors on haze pollution in the current period. ρ and λ, respectively, denote the regression coefficient of spatial lag and spatial error, indicating the spatial spillover effect of haze pollution between cities. $W_{ij}$ denotes a spatial weight matrix, which reflects the spatial connection among different regions. In this paper, the linear distance between each prefecture level city is regarded as the weight which considers the interaction between two cities that are adjacent to the space but not adjacent to each other. $\eta_{i}$, $\nu_{t}$, $\varepsilon_{it}$ respectively denote regional effect, time effect, and random disturbance term. To verify whether there is a non-linear relationship between haze pollution and manufacturing agglomeration, we incorporate the quadratic term of the level of manufacturing agglomeration into the model. This article also incorporates the quadratic item of the economic development level to verify the existence of the environmental Kuznets curve. The model is as follows:

(4) $$\begin{array}{l} \ln I_{it}=\tau \ln I_{i,\left(t-1\right)}+\rho \sum_{j=1}^{N}W_{ij}\ln I_{jt}+\ln\alpha +\beta_{1}\ln{Agglo}_{it}+\beta_{2}{\left(\ln{Agglo}_{it}\right)}^{2}\\ +\gamma_{1}\ln{Pgdp}_{it}+\gamma_{2}{\left(\ln{Pgdp}_{it}\right)}^{2}+\theta \ln{Pop}_{it}+\varphi \ln{Tech}_{it}+\delta \ln X_{it}+\eta_{i}+\nu_{t}+\varepsilon_{it}\\ \varepsilon_{it}=\lambda \sum_{j=1}^{N}W_{ij}\varepsilon_{jt}+\mu_{it} \end{array}$$

### 3.2. Variable Description

Explained variable: the degree of haze pollution. Results of the Ministry of Environmental Protection inspection of air quality show that PM_2.5_ is the primary pollutant of haze pollution in China. This paper therefore uses the annual average concentration value of PM_2.5_ as the proxy variable for urban haze pollution levels. The larger the value of PM_2.5_, the more serious the haze pollution. Since 2013, only a few cities have recorded statistics on PM_2.5_ concentrations and there is a serious lack of historical data. Therefore, haze data are selected from the global average concentration grid data released by the Social Economic Data and Application Center of Columbia University. The specific content of the data is basically consistent with the findings of the Ministry of Environmental Protection on China’s haze problem so the degree of credibility is high. We use ArcGIS software to resolve the data into specific values for the annual average concentration in 285 cities in China from 2003 to 2012.

Core explanatory variables: manufacturing agglomeration index (Agglo). We use the manufacturing employment density to measure the agglomeration index of manufacturing [26]. The larger the value of the manufacturing agglomeration index, the higher the concentration of manufacturing in the region.

Control variables:

Population (Pop). Increases in population lead to larger consumer demand which, in turn, leads to greater emissions of industrial and household pollution. We use the total population of each city at the end of the year to measure the population.

Economic development level (Pgdp). According to the environmental Kuznets hypothesis, the level of economic development has an important impact on environmental pollution [27]. We use per capita GDP to measure economic development levels.

Technical level (Tech). Technological progress can help enterprises adopt cleaner production technologies and improve production efficiency, thus reducing pollutant emissions and improving the environment. We draw on the practice of Xu and Deng [28] and use the capital-labor ratio to measure technical level.

Industrial structure (Stru). In the process of rapid development of industrialization, industrial pollution emission intensity may vary. The energy consumption scale of secondary industry is significantly higher than for other industries. The higher the proportion of secondary industry under the same economic total, the more serious the haze pollution is. Therefore, we use the proportion of added value of secondary industry to GDP to measure the industrial structure of each city.

Transportation (Trans). With the great increase in the number of motor vehicles in China, the emission of vehicle exhaust has also increased significantly, and the CO, NO_X_, and SO_2_ pollution in vehicle exhaust are all important sources of haze. Considering the availability of data, we use the number of civilian vehicles owned in each city to study the effects of transportation on haze pollution.

Foreign direct investment (FDI). On the one hand, foreign enterprises transfer heavily polluting industries to host countries with local governments in those host countries competing to lower environmental regulatory standards in order to attract foreign investment. This results in environmental deterioration [29]. On the other hand, foreign-funded enterprises both introduce cleaner production technologies to their host countries and improve productivity in host country enterprises through technical demonstration and spillover effect. This reduces energy consumption and pollutant emissions, thereby improving environmental pollution [30]. We use the percentage of actual annual of foreign investment to GDP to measure foreign direct investment.

Greening level (Green). Planting green plants in cities can help absorb dust and reduce particulate matter in the air which, in effect, purifies the air. As the greening level can play a certain role in abating urban haze pollution, we use the amount of green coverage in built-up areas to measure the urban greening level.

Central heating (Heat). In winter, the weather is cold in northern China and many cities require central heating. At present, central heating in China is achieved by the burning of coal. This large amount of coal combustion leads to increases in airborne sulfur dioxide and dust particles which in turn aggravate haze pollution. We use the Qinling-Huaihe River line as the boundary of central heating and use a 0–1 dummy variable to measure whether the city has heating in winter [31]. Cities with central heating have a value of 1, and cities without central heating have a value of 0.

### 3.3. Data Sources

To analyze the effect of manufacturing agglomeration on haze pollution, we have selected 2003 to 2012 panel data on 285 prefecture-level and above Chinese cities. The haze data was derived from the global average concentration raster data based on satellite monitoring and released by the Center for Social Economic Data and Applications of Columbia University. The data of other variables were derived from the China Statistical Yearbook (2004–2013), the China City Statistical Yearbook (2004–2013) and the China Regional Economic Statistical Yearbook (2004–2013). Data on the main variables are shown in Table 1.

To verify whether the model has multicollinearity problems, we perform a correlation analysis on all logarithmic variables. Table 2 shows the correlation coefficient matrix of each variable. From Table 2, we can see that there is no high correlation between the explanatory variables. By calculating the VIF (Variance Inflation Factor) of the independent and control variables, we find that the VIF values of all variables are lower than 10, indicating that there is no obvious multicollinearity between the independent variables.

## 4. Empirical Results Analyses

### 4.1. Regression Results and Analysis

The studies of Cheng et al. [31] and Shao et al. [32] showed that haze pollution in China exhibited both a significant global spatial positive correlation and a local spatial agglomeration effect. In our paper, the spatial correlation analysis also finds that haze pollution has a significant global spatial correlation in Chinese cities. There are obvious spatial agglomeration effects in cities with similar degrees of haze pollution. In addition, the high value agglomeration areas and low value agglomeration areas of urban haze pollution in China tend to spatially coalesce, that is, there are significant local spatial agglomeration effects of haze pollution. Because the haze pollution shows spatial correlation and spatial heterogeneity over geographical distances in China, traditional estimation methods may lead to errors. We therefore use the dynamic spatial panel model and, as this model contains both dynamic effects and spatial effects, it makes the estimated results more accurate. In order to highlight the advantages of the dynamic spatial panel model in the regression analysis, we use the following three panel models to regress and then compare the regression results of each model.

Equations (1) and (2) are ordinary panel models. We use the system GMM method proposed by Blundell and Bond [33] to perform regression estimations. This method can overcome the endogeneity problem of explanatory variables in the model. The regression coefficients of the first-order lag of haze pollution in the model are all positive and pass the 1% significance level test. This confirms that haze pollution has a continuous element to it, that is, previous levels have an important effect on haze pollution in current and later periods. The results of AR(1) and AR(2) show that there is no second-order serial correlation in the residuals of the estimation equations and Hansen over-identification test shows that the instrumental variables we selected are reasonable and valid. Equations (3) and (4) are the static spatial panel models, which are estimated by the Maximum Likelihood method (ML) proposed by Elhorst [34]. For the spatial panel model, this paper uses the SAR model as the analysis model after comparing the two Lagrange multipliers and their robustness. Equations (5) and (6) are dynamic spatial panel models, which are estimated by using the spatial system GMM method [35]. By comparing the two Lagrange multipliers and their robustness, the SAR model is used for estimation. The AR(1) and AR(2) tests show that there is no second-order serial correlation in the residuals of the estimated equations, and Hansen over-identification test shows that the instrumental variables we selected are reasonable and valid.

As can be seen from Table 3, the results of the three panel models are basically similar in terms of coefficient sign and significance, indicating that it is appropriate to consider dynamic effects and spatial spillover effects in the analysis of how manufacturing agglomeration affects haze pollution. By comparing the results of the ordinary dynamic panel model and the dynamic spatial panel model, we find that the first-order lag regression coefficients of haze pollution in the ordinary dynamic panel model are significantly higher than those of haze pollution in the dynamic spatial panel model. This is because ordinary dynamic panel models ignore geographic distances and spatial spillover effects, which leads to errors in estimation. The spatial lag regression coefficient of the dynamic spatial panel models is positive and past the 1% significance test, and it can be seen that haze pollution has a significant spatial spillover effect; the results of the dynamic spatial panel model are more accurate.

By comparing the results of the static spatial panel model and the dynamic spatial panel model, it can be seen that the spatial lag regression coefficients of static spatial panel models are significantly higher than those of dynamic spatial panel models. The first-order lag regression coefficient of haze pollution in the dynamic spatial panel models is positive and passes the 1% significance test, indicating that there is a significant dynamic effect of urban haze pollution. Because the static spatial panel models ignore the dynamic effects of haze pollution, it leads to overestimation of spatial lag regression coefficients. We therefore select the regression results of the dynamic spatial panel models to perform the analysis.

From the regression results of Equations (5) and (6), it can be seen that the quadratic coefficient of manufacturing agglomeration is not significant but the coefficient of manufacturing agglomeration is significantly positive. This indicates that manufacturing agglomeration has significantly aggravated haze pollution in China. The reasons are as follows: First, as manufacturing agglomeration occurs, there is an expansion of industrial scale and production capacity; demand for energy increases significantly with a concomitant and rapid increase in pollution. As a result, the urban environmental load exceeds a critical value and leads to the deterioration in environmental quality. Second, most of the industrial agglomeration areas in China are formed under the leadership of government. It is easy to produce continuously repeating construction in the agglomeration areas, which is conducive to neither the technological transformation nor the technological progress of enterprises. In turn, it actually inhibits the development and application of cleaner production technologies, leading to the aggravation of environmental pollution. Third, at present, most of the manufacturing agglomeration areas in China are not, in the real sense, industrial agglomeration. Upstream and downstream industries and their related supporting industries are not closely linked and so fail to achieve the recycling of waste. At the same time, pollutant treatment is dispersed and fails to realize economies of scale.

The coefficient of economic development level is significantly negative, and the coefficient of quadratic term is positive, but it does not pass the significance test indicating that there is no inverted “U” EKC hypothesis between China’s economic development and haze pollution. It shows that Chinese economic development in the current period is conducive to abating haze pollution. The possible reasons are as follows: On the one hand, when economic development reaches a certain point, the mode of economic growth gradually shifts from extensive to intensive; industrial structure is continuously optimized and the proportion of tertiary industry rises rapidly, both of which are conducive to improvements in the environment regionally. On the other hand, as society develops, living standards improve and with these improved living standards, there is a higher demand for environmental quality. At the same time, increases in fiscal revenue allow the government to have sufficient funds to combat haze pollution, thus abating haze pollution to a certain extent.

The coefficient of population on haze pollution is significantly positive at the 1% significance level, indicating that urban population aggravates haze pollution. With the development of the social economy, rapid growth in the urban population not only increases the demand of housing and motor vehicles, but also causes both a shortage of urban land resources and a concentration of excessively dense buildings. This results in road congestion and poor urban air circulation. These all create conditions for haze pollution. On the other hand, the garbage discharged by urban residents contains a large amount of harmful volatile organic compounds. The spread of these organic substances into the air aggravates urban haze pollution.

The technical level has a positive impact on haze pollution emission, and passes the 1% significance test; this was unexpected. It may be due to the fact that industrial agglomeration in China is currently dominated by manufacturing industries which, although tending to improve the levels of production technology, pay less attention to pollution reduction technology in the process of development [32]. Although the per capita capital stock in China has been increasing in recent years, due to the existence of a “crowding-out effect”, the R&D investment of enterprises is mainly used for the improvement of production technology levels, while the R&D expenditure for pollution emission reduction technology is relatively little [28]. With the continuous expansion of enterprise production, the emission of pollutants greatly increases. It follows that, to a certain extent, technical levels aggravate haze pollution in China.

The coefficient of industrial structure on haze pollution is significantly positive at the 1% significance level, showing that industrial structure, measured by the proportion of secondary industry to GDP, aggravates urban haze pollution in China. The proportion of heavy industries in China’s industrial structure is relatively high and high-pollution industries, such as the iron, steel, cement, and chemical industries, are characterized by large energy demand and low utilization efficiency. These industries emit large amounts of noxious gases during the production process, which aggravates haze pollution in China.

The coefficient of transportation is positive and passes the 1% significance test, indicating that transportation exhibits a significant enhancement effect on haze pollution. In recent years, China’s vehicle ownership has risen enormously with carbon based fuel vehicles predominating. As CO, NO_X_, SO_2_, and other gas emissions in vehicle exhaust are the main components of haze, transportation can be counted as an important factor in the aggravation of Chinese haze pollution.

The coefficient of FDI on haze pollution is negative and passes the 5% significance test, indicating that FDI is conducive to abating haze pollution in China. Foreign-funded enterprises promote the improvement of China’s environmental protection technology through both technology spillover effects and demonstration effects. Enterprises often adopt cleaner and more environment-friendly production technologies that reduce their pollution, thus reducing the concentration of haze in Chinese cities.

The coefficient of greening level on haze pollution is negative, indicating that an improvement in levels of greening plays a certain role in improving haze pollution. Most urban greening consists of plants and the planting of green plants helps to absorb dust in the air; this is conducive to removing minute pollutants in the air and purifying it. Cities can improve regional haze pollution by increasing the coverage rate of greening.

The coefficient of central heating on haze pollution is significantly positive at the 1% significance level, indicating that central heating in northern China aggravates haze pollution. At present, most cities in northern China use coal as their principal source of heating in winter. At the present time, most of the environmental dust removal equipment and desulphurization equipment used by the companies that provide heating are not actually put to use, which leads to sulfur dioxide and dust particulates directly discharged into the atmosphere, obviously aggravating local urban haze pollution.

### 4.2. Analysis of Regional Regression Results

The previous analysis shows that the manufacturing industry agglomeration in China has significantly aggravated haze pollution. However, levels of manufacturing agglomeration among the various regions in China differ greatly. Does this difference lead to regional differences in the effects of haze pollution?

In order to compare the effects of manufacturing agglomeration on haze pollution in different regions, we take the Eastern, Central and Western regions as our objects of investigation. The number of cities in Eastern, Central, and Western regions are 101, 100, and 84 respectively. Because the dynamic spatial panel model considers the dynamic and spatial characteristic of haze pollution, the previous analysis also confirms the robustness of the method. Therefore, we use the dynamic spatial panel model for estimations. By comparing the LM test values and the Robust LM test values, we select the SAR model to estimate; Table 4 reports the results of sub-regional regression tests.

The regression results show that the coefficient of manufacturing agglomeration on haze pollution in the three regions are all significantly positive at the 1% significance level indicating that manufacturing agglomeration aggravates haze pollution in the various regions of China. Looking at the influence coefficient of each region, the effect of manufacturing agglomeration on haze pollution is the largest in the Western region, followed by that of the Central region and is least in the Eastern region. It is worth noting that the Eastern region, with the highest concentration of manufacturing agglomeration, has less effect on haze pollution than either the Central and Western regions. The possible reasons are as follows. First, there are many universities and scientific research institutes in the Eastern region. Enterprises in the manufacturing agglomeration areas can improve production efficiency and promote innovation of environmental protection technology by strengthening cooperation with universities and scientific research institutes, all of which is conducive to abating environmental pollution in the agglomeration areas. Second, in the Eastern region, manufacturing agglomeration developed earlier and residents in the agglomeration areas have a higher standard of living. They also have higher requirements and expectations regarding environmental quality, which forces the government to increase investment in environmental governance; this is conducive to improving environmental pollution in agglomeration areas. Finally, the pattern of agglomeration development for manufacturing industries in the Eastern region is gradually changing from labor-intensive and resource-intensive to technology-intensive; this is conducive to improving production efficiency and reducing the emission of pollutants in the agglomeration areas. In order to speed up economic development, the Central and Western regions often lower their environmental regulation standards. This causes a large number of labor-intensive industries from the Eastern region to move there and thus the agglomeration of labor-intensive industries leads to the further aggravation of haze pollution in Central and Western regions.

### 4.3. Robustness Test

In order to further enhance the robustness of the above regression results, we use location entropy to measure the degree of manufacturing agglomeration in various cities in China [21,36]. Location entropy is a common method used to measure manufacturing agglomeration, which can better analyze the degree of manufacturing agglomeration from a regional perspective. The data and meteorology analysis software required for the robustness test are consistent with the previous text. Table 5 gives results of the overall robustness test and the sub-regional robustness test.

By observing the results of robustness regression, when the manufacturing agglomeration index is measured by location entropy, the coefficient of manufacturing agglomeration is significantly positive at the national level, which indicates that manufacturing agglomeration has significantly aggravated haze pollution in China. The coefficient of manufacturing agglomeration on haze pollution in the three regions are all significantly positive at the 1% significance level, comparing the influence coefficient of each region, the effect of manufacturing agglomeration on haze pollution is the largest in the Western region, followed by that of the Central region and is least in the Eastern region, this result is consistent with the regression results in Table 4. The coefficient symbols of other variables are consistent with the previous estimation results. There is, however, a certain difference between the magnitude of the values and the level of significance, further indicating that the regression results of the above models are robust.

## 5. Conclusions

This paper analyzes the effect of manufacturing agglomeration on haze pollution. The results show that manufacturing agglomeration significantly aggravates haze pollution when economic development levels, population quantity, technological levels, industrial structure, transportation, foreign direct investment, and greening levels are all stable. The rapid growth of population, industrial structure, as measured by the proportion of second industry to GDP, and increasing vehicle ownership all aggravate haze pollution. In northern China, central heating in winter also aggravates the haze pollution to a certain extent. Improvements in economic development levels, foreign direct investment, and greening levels are conducive to reductions in haze pollution. Regional comparative analysis shows that there are regional differences in the effects of manufacturing agglomeration on haze pollution. The effect of manufacturing agglomeration on haze pollution is largest in the Western region, followed by the Central region, and least in the Eastern region. In view of the above conclusions, the recommendations of this paper are as follows:

China should give full play to the technology and knowledge spillover effects of manufacturing agglomeration and promote innovation in both production and pollution treatment technologies. First, China should encourage competition and cooperation for enterprises in the agglomeration regions, promote knowledge spillover and technological innovation, improve both production efficiency and pollution treatment technology levels and reduce the emission of pollutants. Second, China should encourage manufacturing enterprises in the agglomeration regions to accept the more advanced environmental technology and management experience of foreign enterprises and improve efficiency in environmental governance. Finally, China should use higher environmental regulation standards to force enterprises to improve both production technology and energy efficiency; this ought to improve environmental quality.

(2) China should guide manufacturing agglomerations in a scientific and rational way and give full play to the advantages in energy conservation and emissions reduction. On the one hand, the industrial chain, with upper and lower reaches and related supporting industries, should be built around a leading industry in order to realize the recycling of the various raw materials in the production process, thus reducing the emission and surplus of the pollutants. On the other hand, China should strengthen the environmental protection infrastructure in the manufacturing agglomeration regions by centralizing pollution management; this should improve efficiency in the treatment of pollutants.

(3) China should accelerate the transformation and upgrading of manufacturing industries in agglomeration regions and encourage the green development of manufacturing agglomerations. For the Eastern region, China should promote the development of clean and high added value enterprises in the agglomeration regions, closely monitor and encourage technological innovation within manufacturing industries and lead and promote the transformation and upgrading of manufacturing industries as regards scientific and technological innovation. For the Central and Western regions, when enticing manufacturing enterprises from the Eastern region, manufacturing enterprises should both be located following a reasonable plan and according to their own development needs. They should also improve the standards of environmental regulations in the agglomeration regions so as to avoid the tragedy of “treatment after pollution”. Secondly, China should encourage enterprises in the agglomeration regions to carry out technological innovation, reform traditional production processes, improve both production efficiency and the level of environmental protection technology and reduce polluting emissions.

## Figures and Tables

**Table 1:** Descriptive statistics of major variables.

Variable	Sample Size	Mean	Std. Dev.	Min	Max
lnI	2850	3.705	0.553	1.708	4.687
lnAgglo	2850	1.687	1.526	−3.811	6.450
lnPop	2850	5.838	0.691	2.795	8.115
lnPgdp	2850	9.823	0.785	4.595	12.12
lnTech	2850	3.401	0.784	−0.541	5.536
lnStru	2850	3.894	0.282	2.086	4.511
lnTrans	2850	2.487	1.171	−6.742	12.746
lnFDI	2850	−6.556	1.471	−12.995	−3.092
lnGreen	2850	3.494	0.458	−1.022	5.957
Heat	2850	0.435	0.496	0	1

**Table 2:** Correlation Analysis and VIF Test.

	VIF	lnI	lnAgglo	lnPop	lnPgdp	lnTech	lnStru	lnTrans	lnFDI	lnGreen	Heat
lnI		1.000									
lnAgglo	2.57	0.448	1.000								
lnPop	2.43	0.444	0.228	1.000							
lnPgdp	5.15	0.109	0.533	−0.089	1.000						
lnTech	2.50	0.246	0.136	0.069	0.670	1.000					
lnStru	1.30	0.219	0.350	−0.097	0.356	0.178	1.000				
lnTrans	3.38	0.291	0.455	0.528	0.587	0.485	0.103	1.000			
lnFDI	1.66	0.238	0.534	0.220	0.392	0.246	0.097	0.344	1.000		
lnGreen	1.34	0.184	0.347	0.089	0.414	0.285	0.233	0.281	0.392	1.000	
Heat	1.27	−0.079	−0.159	−0.140	0.109	−0.024	0.069	0.091	−0.241	−0.100	1.000

**Table 3:** Overall Regression Results.

Type	Ordinary Dynamic Panel Models		Static Space Panel Models		Dynamic Space Panel Models	
	Equation (1)	Equation (2)	Equation (3)	Equation (4)	Equation (5)	Equation (6)
τ	0.734 *** (9.30)	0.741 *** (9.52)			0.175 *** (5.16)	0.182 *** (5.39)
ρ			0.314 *** (8.76)	0.311 *** (8.42)	1.93 × 10^−6^ *** (3.60)	1.90 × 10^−6^ *** (3.54)
lnAgglo	0.068 *** (4.03)	0.082 *** (4.26)	0.056 *** (6.11)	0.052 *** (4.52)	0.131 *** (10.09)	0.139 *** (10.28)
(lnAgglo)^2^		0.005 (0.92)		0.002 (0.85)		−0.003 (−0.98)
lnPgdp	−0.023 *** (−2.73)	−0.030 *** (−3.32)	−0.024 *** (−2.72)	−0.029 *** (−3.22)	−0.291 *** (−10.01)	−0.296 *** (−10.12)
(lnPgdp)^2^		−0.012 (−1.27)		0.011 (1.21)		0.004 (0.58)
lnPop	0.090 ** (2.22)	0.111 ** (2.59)	0.057 * (1.83)	0.061 * (1.95)	0.157 *** (9.90)	0.156 *** (9.72)
lnTech	0.089 *** (2.73)	0.107 *** (3.05)	0.095 *** (15.45)	0.100 *** (15.89)	0.221 *** (12.44)	0.219 *** (12.23)
lnStru	0.056 (1.29)	0.059 (1.14)	0.064 *** (5.31)	0.066 *** (5.43)	0.182 *** (6.76)	0.176 *** (6.32)
lnTrans	0.044 ** (1.87)	0.046 ** (2.06)	0.069 *** (5.81)	0.070 *** (5.71)	0.034 *** (2.93)	0.033 *** (2.80)
lnFDI	−0.002 (−0.40)	−0.006 (−0.86)	−0.010 (−1.47)	−0.009 (−1.41)	−0.013 ** (−1.57)	−0.014 ** (−1.16)
lnGreen	−0.008 (−0.71)	−0.006 (−0.42)	−0.024 (−1.29)	−0.024 (−1.25)	−0.030 ** (−1.94)	−0.031 ** (−2.00)
Heat	0.032 *** (3.28)	0.028 ** (2.18)	0.038 *** (3.42)	0.027 ** (2.20)	0.033 *** (3.36)	0.028 ** (2.20)
Adj-R^2^			0.487	0.489		
Obs	2565	2565	2850	2850	2565	2565
LM_Lag test			3410.68	3413.17	4235.21	4264.14
Robust LM_Lag test			550.65	546.43	929.65	953.06
LM_Error test			3052.70	3034.99	3772.64	3744.14
Robust LM_Error test			467.08	433.07	908.64	924.61
AR(1) test	(0.016)	(0.019)			(0.010)	(0.012)
AR(2) test	(0.251)	(0.243)			(0.273)	(0.284)
Hansen test	(0.998)	(0.998)			(0.999)	(0.999)

Figures in parentheses are *t* values; ***, **, and * denote a significance of 1%, 5% and 10%, respectively.

**Table 4:** Sub-regional test results.

Type	East Areas		Central Areas		West Areas	
	Equation (7)	Equation (8)	Equation (9)	Equation (10)	Equation (11)	Equation (12)
τ	0.467 *** (9.58)	0.461 *** (10.40)	0.329 *** (5.25)	0.358 *** (5.75)	0.062 * (1.54)	0.068 * (1.67)
ρ	1.50 × 10^−5^ *** (3.30)	1.33 × 10^−5^ *** (2.98)	7.07 × 10^−6^ *** (1.62)	7.38 × 10^−6^ *** (1.23)	6.03 × 10^−6^ *** (1.53)	7.61 × 10^−6^ *** (1.31)
lnAgglo	0.144 *** (9.88)	0.151 *** (10.16)	0.155 *** (10.27)	0.171 *** (12.17)	0.161 *** (11.10)	0.175 *** (12.32)
(lnAgglo)^2^		−0.011 (−1.87)		−0.006 (−1.62)		−0.002 (−0.08)
lnPgdp	−0.197 *** (−5.35)	−0.186 *** (−4.85)	−0.104 *** (−4.24)	−0.102 *** (−4.16)	−0.094 ** (−2.29)	−0.074 * (−1.90)
(lnPgdp)^2^		0.016 (1.24)		0.015 (1.20)		−0.009 (−0.75)
lnPop	0.058 *** (2.91)	0.060 *** (3.34)	0.051 *** (3.33)	0.047 *** (3.02)	0.287 *** (9.25)	0.295 *** (9.21)
lnTech	0.301 *** (10.92)	0.308 *** (11.40)	0.088 *** (4.88)	0.082 *** (4.53)	0.214 *** (5.26)	0.220 *** (5.41)
lnStru	0.030 * (1.16)	0.041 * (1.49)	0.235 *** (4.65)	0.215 *** (4.33)	0.104 * (1.77)	0.127 ** (2.15)
lnTrans	0.060 ** (2.45)	0.054 ** (2.17)	0.018 * (1.86)	0.018 * (1.93)	0.147 *** (6.38)	0.169 *** (7.25)
lnFDI	−0.062 *** (−4.72)	−0.068 *** (−5.30)	−0.031 *** (−4.03)	−0.030 *** (−3.83)	−0.023 ** (−2.00)	−0.029 ** (−2.55)
lnGreen	−0.006 (−0.17)	−0.009 (−0.40)	−0.026 * (−1.25)	−0.026 * (−1.10)	−0.017 (−0.80)	−0.016 (−0.67)
Heat	0.484 *** (10.17)	0.403 *** (8.28)	0.200 *** (7.52)	0.183 *** (6.78)	0.050 (0.83)	0.053 (0.91)
logL	76.65	82.19	314.87	307.60	251.01	252.29
Obs	909	909	900	900	756	756

Figures in parentheses are t values; ***, **, and * denote a significance of 1%, 5% and 10%, respectively.

**Table 5:** Robustness test results.

Type	National Level		East Areas		Central Areas		West Areas	
	Equation (13)	Equation (14)	Equation (15)	Equation (16)	Equation (17)	Equation (18)	Equation (19)	Equation (20)
τ	0.272 *** (7.20)	0.277 *** (7.45)	0.571 *** (11.49)	0.592*** (12.09)	0.434 *** (6.65)	0.446 *** (6.86)	0.119 *** (2.63)	0.115 *** (2.48)
ρ	2.35 × 10^−6^ *** (4.16)	2.29 × 10^−6^ *** (4.05)	1.65 × 10^−5^ *** (3.72)	1.69 × 10^−5^ *** (3.58)	8.07 × 10^−6^ *** (8.13)	8.46 × 10^−6^ *** (8.29)	3.03 × 10^−6^ (0.74)	3.13 × 10^−6^ (0.77)
lnAgglo	0.067 *** (2.98)	0.060 *** (2.84)	0.077 *** (3.19)	0.076 *** (3.17)	0.149 *** (3.26)	0.147 *** (3.20)	0.164 *** (3.38)	0.159 *** (3.17)
(lnAgglo)^2^		−0.004 (−0.40)		−0.003 (−0.39)		−0.017 (−0.91)		−0.014 (−0.86)
lnPgdp	−0.072 *** (−3.55)	−0.076 *** (−3.22)	−0.099 *** (−2.94)	−0.093 ** (−2.83)	−0.092 ** (−2.79)	−0.085 ** (−2.29)	−0.076 * (−1.87)	−0.073 * (−1.73)
(lnPgdp)^2^		0.001 (0.01)		0.002 (0.09)		0.023 (1.62)		−0.011 (−0.09)
lnPop	0.158 *** (9.73)	0.163 *** (9.98)	0.109 *** (3.62)	0.074 ** (2.46)	0.077 *** (4.44)	0.076 *** (4.23)	0.328 *** (9.53)	0.337 *** (9.74)
lnTech	0.117 *** (6.84)	0.113 *** (6.61)	0.211 *** (8.17)	0.202 *** (7.93)	0.044 ** (2.42)	0.043 *** (2.33)	0.046 (1.16)	0.037 (0.91)
lnStru	0.239 *** (8.67)	0.244 *** (8.83)	0.232 *** (4.39)	0.271 *** (5.19)	0.054 * (1.86)	0.051 * (1.74)	0.226 *** (3.62)	0.209 *** (3.30)
lnTrans	0.171 *** (3.57)	0.161 *** (3.29)	0.107 *** (4.44)	0.092 *** (3.86)	0.037 *** (3.39)	0.036 *** (3.37)	0.124 *** (4.97)	0.125 *** (4.90)
lnFDI	−0.051 * (−1.92)	−0.042 * (−1.55)	−0.049 *** (−3.69)	−0.030 ** (−2.27)	−0.014 * (−1.61)	−0.016 * (−1.84)	−0.007 (−0.54)	−0.007 (−0.56)
lnGreen	−0.046 *** (−3.01)	−0.044 *** (−2.84)	−0.003 (−0.09)	−0.024 (−0.67)	−0.008 (−0.33)	−0.002 (−0.06)	−0.037 (−1.45)	−0.037 (−1.46)
Heat	0.094 *** (10.03)	0.094 *** (9.96)	0.473 *** (9.34)	0.426 *** (8.35)	0.136 *** (4.42)	0.124 *** (4.02)	0.106 *** (3.92)	0.104 *** (3.86)
logL	496.56	493.36	197.13	202.19	243.21	239.64	298.00	295.65
Obs	2565	2565	909	909	900	900	756	756

Figures in parentheses are t values; ***, **, and * denote a significance of 1%, 5%, and 10%, respectively.
